# Clipped Axillary Node as a Potential Surrogate for Overall Axillary Nodal Status in Inflammatory Breast Cancer Patients after Neoadjuvant Chemotherapy

**DOI:** 10.1245/s10434-024-15796-7

**Published:** 2024-08-09

**Authors:** Kush R. Lohani, Tanya L. Hoskin, Saba Yasir, Carrie A. Olson, Judy C. Boughey, Tina J. Hieken, Amy C. Degnim

**Affiliations:** 1 Division of Breast and Melanoma Surgical Oncology, Department of Surgery, Mayo Clinic, Rochester, MN USA; 2 Division of Clinical Trials and Biostatistics, Department of Quantitative Health Sciences, Mayo Clinic, Rochester, MN USA; 3 Department of Pathology, Mayo Clinic, Rochester, MN USA

**Keywords:** Inflammatory breast cancer, Axillary nodal dissection, Clipped axillary node, False negative rate

## Abstract

**Background:**

Axillary lymph node dissection is the current standard for management of the axilla in inflammatory breast cancer (IBC). The present study aims to determine whether the initially positive node identified by clip placement accurately represents the overall nodal status of axilla after neoadjuvant chemotherapy (NAC) in IBC.

**Patients and Methods:**

A retrospective study was conducted on patients with IBC who underwent operation (2014–2023). For patients with IBC who had clip placement in a positive axillary node at diagnosis, operative notes, specimen radiographs, and pathology reports were reviewed to confirm final pathologic status of clipped nodes.

**Results:**

In total, 92 patients with IBC (90 cN+) were identified (median age 54 years, 78% invasive ductal, 10% invasive lobular, and 12% mixed); 81 (90%) were biopsy-proven cN+, with a clip placed in the positive node for 62/81 (77%). All patients were treated with NAC and axillary surgery with median 19 (range 4–49) nodes removed. Among 28 (out of 56) patients with retrieved clipped nodes that were pathologically negative (ypN0), only 1 had an additional positive node with micrometastasis for a false negative rate of 4% (95% CI 1–19%). Conversely, 3/3 patients with isolated tumor cells (ITCs) only in the clipped node had additional axillary disease (ITCs in 1, macrometastasis in 2), and 20/23 (87%) of patients with pathologically positive clipped node (micrometastasis or greater) had additional positive nodes [19/20 (95%) with macrometastasis].

**Conclusions:**

The clipped biopsy-positive axillary node in IBC accurately represented the post-NAC overall axillary nodal status. ITCs post-NAC should be considered positive as an indicator of additional nodes with metastasis.

**Supplementary Information:**

The online version contains supplementary material available at 10.1245/s10434-024-15796-7.

Inflammatory breast cancer (IBC) is the most aggressive form of breast cancer (BC), with nodal disease in up to 85% and distant metastasis in 30% at presentation.^1^ The current standard of care is multimodality treatment comprising neoadjuvant chemotherapy (NAC), modified radical mastectomy, and adjuvant chemoradiotherapy. However, more than 50% of patients with IBC develop lymphedema following this treatment regime.^2^ While surgical axillary clearance is the current standard, recent research shows that outcomes of IBC are mainly driven by tumor subtype^3^ and pathological complete response (pCR), facilitated by chemotherapeutic advances and anti-HER2 targeted therapies.^4^ Hence, deescalating ALND in selected patients with IBC with nodal complete pathological response (ypN0) may be a possibility. However, finding an accurate minimally invasive axillary staging procedure in IBC has been technically challenging.

Early reports showed a very high false negative rate (FNR) of 25% in a small series of patients with IBC undergoing sentinel node biopsy,^5^ and more recent series reported failure of dual tracer sentinel lymph node biopsy (SLNB) mapping in up to 75% of patients with IBC.^6^ In the non-IBC setting, de-escalation of axillary surgical intervention has gained importance, from avoiding ALND in a clinically node negative (cN0) breast cancer (BC) with a positive SLNB^7–9^ to establishing the feasibility of post-NAC SLNB in clinically node positive patients.^10^ Furthermore, targeted SLNB post-NAC by retrieval of the clipped node has been shown to decrease false negative rate (FNR) further to an acceptable value of 6.8% in patients who do not have IBC.^11^ The purported reason for lymphatic mapping failure in IBC is blocked lymphatics, either due to tumor obstruction or to post-NAC scarring. Therefore, a minimally invasive axillary approach in IBC that does not rely on successful lymphatic mapping with tracers might hold more promise for success. Retrieving a clipped positive axillary node in IBC post-NAC provides a technical solution for targeted axillary staging that does not rely on lymphatic mapping and that might accurately reflect the pathologic status of the remaining axillary nodal basin. Therefore, we hypothesized that the pathologic status of a clipped positive axillary node in IBC will correlate with overall pathologic axillary nodal status at axillary dissection, and we undertook a retrospective review of IBC cases at our institution to investigate this hypothesis.

## Patients and Methods

Ater institutional review board approval, a retrospective study was conducted in patients diagnosed with IBC from January 2014 through December 2023. All patients with IBC (cT4d cN0-3 M0-1) who underwent axillary nodal surgery post-NAC were included in the analysis. Patient data were abstracted from the electronic medical record on demographics, clinical tumor subtype and staging, imaging, NAC, surgery, and histopathological findings. Patients were classified as node positive if they had positive fine-needle aspiration (FNA) or positive lymph node core biopsy prior to the start of neoadjuvant therapy (biopsy-proven cN+), or if they met clinical/imaging criteria and were judged to have lymph node positive disease by the treating physician (cN+ without a pre-treatment positive biopsy or FNA). The latter group did include a small number of patients (*n *= 4) who were deemed to be clinically-node positive despite a negative FNA/biopsy result.^12,13^ These patients had pathologic palpable adenopathy and corroborating suspicious nodes on imaging [positron emission tomography (PET) in 3, magnetic resonance imaging (MRI) in 1] and were judged by the clinical team to be clinically node positive.

Nodal response to NAC was recorded from physical exam, available imaging modalities, and the clinical assessment of the treating surgeon (which incorporates physical exam and imaging findings). In our clinical practice, dedicated breast radiologists routinely comment on response to chemotherapy in both breast and lymph nodes. Specific language in the imaging report that described “complete response” or “normalization” of lymph nodes was classified as a complete response. Post-NAC clinical nodal status was classified as ycN0 or ycN+ using both clinical examination and imaging findings as follows: patients were classified as ycN0 if both the physical exam and all available post-NAC axillary imaging were negative, or as ycN+ if physical exam or any imaging was positive.

Information on any preoperative localization of the clipped node with I-125 seeds was also abstracted. Surgical retrieval of the clipped axillary node was confirmed by detailed review of operative notes, specimen radiographs, and/or pathology reports. If the pathologic status of the clipped node still was unclear, original pathology slides of the axillary nodes were re-reviewed with an expert breast pathologist to look for biopsy site changes and to confirm presence or absence of metastasis in the clipped node. Efficacy of post-NAC imaging (ultrasound, magnetic resonance imaging, positron emission tomography) in predicting nodal status were also determined and compared with that of clipped nodal status in relation to the final overall pathological nodal status. The size of nodal metastasis was characterized as macrometastasis (tumor deposit > 2 mm), micrometastasis (approximately 200 tumor cells, tumor deposit larger than 0.2 mm, but none larger than 2.0 mm), or isolated tumor cells ITCs (malignant cell clusters no larger than 0.2 mm). The American Joint Committee on Cancer (AJCC) staging criteria were used to classify final pathological nodal staging on the basis of the total number of positive nodes. Per AJCC criteria, lymph nodes with ITCs only were classified as negative in post-NAC setting (ypN0i+). However, post-NAC isolated tumor cells (ITCs) in the clipped node were reported separately from strictly ypN0i+ clipped nodes for descriptive purposes.

Tumors were defined as hormone-receptor positive (HR+) if they were estrogen-receptor and/or progesterone-receptor positive at ≥ 1%. HER2-status was defined as positive if 3+ on immunohistochemistry (IHC) or amplified on fluorescent in situ hybridization (FISH) and as negative if 0 to 1+ on IHC or not amplified on FISH. Approximated tumor biologic subtypes were defined as HR+/HER2−, HER2+ (including HR+ and HR−), and triple-negative breast cancer (TNBC).

Nodal pathologic complete response (pCR) was defined as ypN0/N0i+, and breast pCR was defined as no residual invasive breast tumor (ypT0/Tis). A patient was classified as having total pCR if they had both nodal and breast pCR and as not having total pCR if there was residual invasive disease in either lymph nodes (ypN1mi or greater) or breast (ypT1mi or greater).

### Statistical Analysis

Patients with a clip placed in a biopsy-positive lymph node (“clipped positive node” group) were compared with cN+ patients without a clipped biopsy-positive lymph node (“no clipped positive node” group) using Wilcoxon rank-sum tests for continuous variables and Fisher’s exact tests for categorical variables. The “no clipped positive node” group included patients who were (a) biopsy proven cN+ but without clip placement, (b) cN+ based on clinical/imaging criteria only with no pre-treatment FNA or lymph node biopsy performed, (c) FNA/biopsy-negative with no clip placement but deemed cN+ by clinical/imaging criteria, or (d) FNA/biopsy-negative with a clip placed in the biopsy-negative lymph node. Diagnostic performance characteristics, including sensitivity, specificity, and FNR, of the clipped biopsy-positive node and of post-NAC imaging modalities were calculated using the final pathologic examination of all removed axillary nodes as the gold standard. For imaging modalities, the test was considered positive for residual nodal disease if it showed partial or no response in the axilla and as negative for residual disease if it showed a complete response in the axilla. Confidence intervals (CI) for binomial proportions were calculated using the Wilson score method. Analysis was performed using SAS (SAS Institute Inc., Cary, NC, Version 9.4), and *p*-values < 0.05 were considered statistically significant.

## Results

### Study Population and Patient Characteristics

**Fig. 1:**
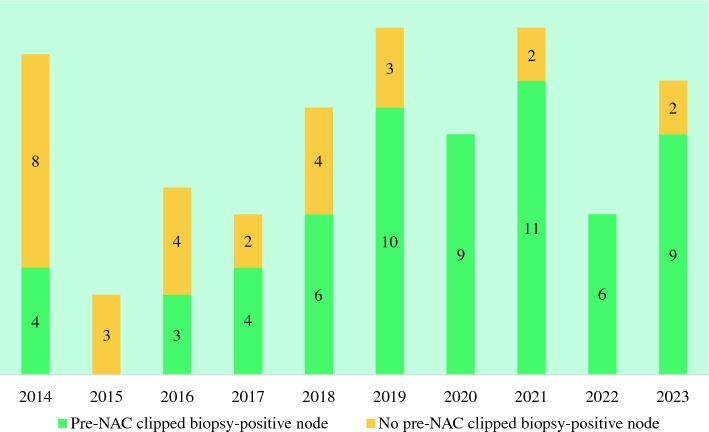
Number of patients with inflammatory breast cancer who had a clip placed in the setting of a biopsy positive axillary node at presentation

We studied 92 patients with IBC, including 5 with cM1 oligometastatic disease who had good response to NAC and multidisciplinary decision to proceed with surgery and ALND. At the time of diagnosis, 98% (90/92) patients were classified as clinically node positive (cN+). There were two cN0 patients, 1 biopsy negative who was ypN1 at ALND and the other had no FNA/biopsy and was ypN0 at ALND. Of the 90 cN+ patients, 9 (10%) were classified on the basis of clinical and imaging criteria only (no positive FNA/biopsy) and 81 (90%) were biopsy proven cN+, with a clip placed in the positive node for 62/81 (77%). The majority of cN+ patients with IBC overall had axillary clips placed (69%), with increasing proportion from 2014 (33%) to 2020 (100%) and remaining above 80% through 2023 (Fig. 1). The 28 patients without a biopsy-positive clipped axillary node included 19 patients with a positive FNA/biopsy but without clip placement, 5 patients judged clinically node positive without pre-NAC axillary FNA/biopsy, and 4 patients who were FNA/biopsy negative but still considered cN+ on the basis of clinical/imaging criteria (2 with clip placement in the biopsy-negative node and 2 without clip placement). Of the 81 cN+ patients with positive pre-NAC axillary biopsy, 37 were diagnosed at our institution and 44 were initially diagnosed elsewhere before being referred to our institution; patients initially diagnosed elsewhere were more likely to have clip placed (91% versus 59%, *p *= 0.001), but this was driven by the early years of the cohort before our practice had fully shifted to routine clipping of positive nodes; during the latter years of the cohort (2019–2023), 96% of biopsy-positive nodes were clipped, including 95% of those diagnosed at our institution and 96% of those initially diagnosed elsewhere.

Characteristics of the 90 cN+ patients are summarized in Table 1, along with breakdown of features in the subgroups who did and did not have axillary clips placed. Overall, most features were similar between the two groups. Median age was 54 years overall (range 22–87 years). The majority of patients were white (90%), reflective of the regional population. Regarding tumor histology, 78% were invasive ductal, 10% were invasive lobular, and 12% were mixed invasive ductolobular. All patients were clinically node positive: 43% cN1, 12% cN2, and 44% cN3. Tumor biological subtype overall was 40% HR+/HER2−, 37% HER2+, and 23% TNBC. The majority were high grade (73%), with lymphovascular invasion in 40%. All patients received neoadjuvant chemotherapy, with taxanes in 97%, anthracyclines in 8%, and pembrolizumab in 9%.

**Table 1:** Demographic and clinicopathologic characteristics of *n* = 90 cN+ inflammatory BCs overall and comparing patients with versus without a pre-treatment biopsy-positive clipped node

	Total (*N* = 90)	Clipped biopsy+ node (*N* = 62)	No biopsy+ clipped node (*N* = 28)	*p*-Value
Age				0.06
Median	54.0	55.0	51.0	
Range	22.0, 83.0	22.0, 83.0	24.0, 79.0	
IQR	46.0, 63.0	49.0, 64.0	39.5, 59.5	
Race, *n* (%)				0.35
White	81 (90.0%)	54 (87.1%)	27 (96.4%)	
American Indian/Alaska Native	3 (3.3%)	3 (4.8%)	0 (0.0%)	
Asian	4 (4.4%)	4 (6.5%)	0 (0.0%)	
Other	2 (2.2%)	1 (1.6%)	1 (3.6%)	
Ethnicity, *n* (%)				> 0.99
Hispanic or Latino	1 (1.1%)	1 (1.6%)	0 (0.0%)	
Not Hispanic or Latino	88 (97.8%)	60 (96.8%)	28 (100%)	
Unknown	1	1	0	
Tumor Histology, *n* (%)				0.92
Invasive ductal carcinoma	70 (77.8%)	49 (79.0%)	21 (75.0%)	
Invasive lobular carcinoma	9 (10.0%)	6 (9.7%)	3 (10.7%)	
Mixed invasive ductolobular				
Carcinoma	11 (12.2%)	7 (11.3%)	4 (14.3%)	
Clinical nodal status at presentation, *n* (%)				0.26
cN1	39 (43.3%)	25 (40.3%)	14 (50.0%)	
cN2	11 (12.2%)	10 (16.1%)	1 (3.6%)	
cN3	40 (44.4%)	27 (43.5%)	13 (46.4%)	
Axilla level, *n* (%)				0.12
1	30 (33.3%)	25 (40%)	5 (17.9%)	
2	33 (36.7%)	20 (32.3%)	13 (46.4%)	
3	27 (30.0%)	17 (27.4%)	10 (35.7%)	
Metastasis at presentation, *n* (%)				> 0.99
cM0	85 (94.4%)	58 (93.5%)	27 (96.4%)	
cM1	5 (5.6%)	4 (6.5%)	1 (3.6%)	
Tumor subtype, *n* (%)				0.74
HR+ and HER2−	36 (40.0%)	23 (37.1%)	13 (46.4%)	
HER2+	33 (36.7%)	24 (38.7%)	9 (32.1%)	
TNBC	21 (23.3%)	15 (24.2%)	6 (21.4%)	
Grade of tumor, *n* (%)				0.45
2	24 (26.7%)	15 (24.2%)	9 (32.1%)	
3	66 (73.3%)	47 (75.8%)	19 (67.9%)	
Lymphovascular invasion, *n* (%)				> 0.99
No	52 (59.8%)	37 (59.7%)	15 (60.0%)	
Yes	35 (40.2%)	25 (40.3%)	10 (40.0%)	
Missing	3	0	3	
Ki67				0.11
Median	40.0	39.6	45.9	
Range	1.0, 98.0	1.0, 60.0	14.7, 98.0	
IQR	30.0, 50.8	24.2, 47.0	33.3, 74.9	
NAC-anthracycline-based, *n* (%)				0.002
No	16 (17.8%)	16 (25.8%)	0 (0.0%)	
Yes	74 (82.2%)	46 (74.2%)	28 (100%)	
NAC-taxanes, *n* (%)				> 0.99
No	3 (3.3%)	2 (3.2%)	1 (3.6%)	
Yes	87 (96.7%)	60 (96.8%)	27 (96.4%)	
NAC-pembrolizumab, *n* (%)				> 0.99
No	82 (91.1%)	56 (90.3%)	26 (92.9%)	
Yes	8 (8.9%)	6 (9.7%)	2 (7.1%)	
Clinical nodal status (post-NAC), *n* (%)				0.81
ycN0	60 (68.2%)	40 (66.7%)	20 (71.4%)	
ycN+	28 (31.8%)	20 (33.3%)	8 (28.6%)	
Missing	2	2	0	
Breast surgery, *n* (%)				
Mastectomy	90 (100.0%)	62 (100.0%)	28 (100.0%)	
Axillary surgery, *n* (%)				> 0.99
Sentinel node biopsy only	1 (1.1%)	1 (1.6%)	0 (0.0%)	
Axillary nodal dissection	89 (98.9%)	61 (98.4%)	28 (100.0%)	
Total nodes removed				0.08
Median	19.0	18.5	21.5	
Range	4.0, 49.0	4.0, 49.0	7.0, 42.0	
IQR	12.0, 25.0	12.0, 23.0	15.5, 27.5	
Pathological nodal status, *n* (%)				0.48
ypN0/N0i+	46 (51.1%)	32 (51.6%)	14 (50.0%)	
ypN1	16 (17.8%)	13 (21.0%)	3 (10.7%)	
ypN2	15 (16.7%)	10 (16.1%)	5 (17.9%)	
ypN3	13 (14.4%)	7 (11.3%)	6 (21.4%)	
Extranodal extension,* n* (%)				0.45
No	64 (71.1%)	46 (74.2%)	18 (64.3%)	
Yes	26 (28.9%)	16 (25.8%)	10 (35.7%)	
Nodes with treatment effect, *n* (%)				0.25
No	16 (17.8%)	9 (14.5%)	7 (25.0%)	
Yes	74 (82.2%)	53 (85.5%)	21 (75.0%)	
Pathological tumor status,* n* (%)				0.47
ypT0	31 (34.4%)	23 (37.1%)	8 (28.6%)	
ypTis	7 (7.8%)	6 (9.7%)	1 (3.6%)	
ypT1	19 (21.1%)	14 (22.6%)	5 (17.9%)	
ypT2	5 (5.6%)	4 (6.5%)	1 (3.6%)	
ypT3	11 (12.2%)	6 (9.7%)	5 (17.9%)	
ypT4	17 (18.9%)	9 (14.5%)	8 (28.6%)	
Total pCR, *n* (%)				0.64
Yes	33 (36.7%)	24 (38.7%)	9 (32.1%)	
No	57 (63.3%)	38 (61.3%)	19 (67.9%)	

At surgery, all underwent modified radical mastectomy, except for a single patient in the clipped-node group where seed-localized targeted SLNB was done with retrieval of the clipped node due to her comorbidity and age. Overall, seed localization was performed for 13% (8/62) patients with a clipped biopsy-positive node, and the median number of nodes removed at surgery was 19.

### Clinical Response and Pathological Nodal Status Post-NAC in IBC

Post-NAC axillary imaging included ultrasound in 50/90 (56%), MRI in 50/90 (56%), and PET in 38/90 (42%). Among those with post-NAC imaging available for each modality, complete nodal response to NAC by imaging reports was 60% (30/50) by axillary ultrasound (US), 62% (31/50) by MRI, and 79% (30/38) by PET. Assessment of nodal status by individual imaging modalities is presented in Supplementary Table 1. In total, post-NAC clinical node category (ycN status) as classified by our algorithm (combining clinical exam and imaging findings) was missing for 2 patients and classifiable in 88/90 (98%), who were 68% (60/88) ycN0 and 32% (28/88) ycN+. Clinical nodal status post-NAC did not differ significantly between patients with and without a clip placed (33% ycN+ versus 29% ycN+, respectively, *p *= 0.81), Table 1.

On surgical pathology, half of patients (46/90) had nodal pCR, with similar percentages in patients with and without clipped biopsy-positive node (52% versus 50%, *p* > 0.99). This included one patient in the clipped node group who had two nodes (the clipped node and one additional) each with ITCs only. Nodal pCR was more frequent among HER2+ patients (25/33 or 76%) than HR+/HER2− (14/36 or 39%) and TNBC (7/21 or 33%), *p *= 0.002. Breast pCR (defined as ypT0/Tis) was observed in 38/90 (42%) overall, and total pCR (ypT0/Tis, ypN0/N0i+) was observed in 33/90 (37%). Post-NAC clinical (ycN) nodal status as documented and classified from the clinical record was not significantly associated with ypN status: patients classified as ycN0 were 57% ypN0 and 43% ypN+, while patients classified as ycN+ were 39% ypN0 and 61% ypN+ (*p *= 0.17). Pathology findings in the clipped nodes are presented in Table 2.

**Table 2:** Inflammatory BCs with clipped biopsy-positive node with confirmed retrieval (*n* = 56)

Pathologic findings in clipped biopsy-positive node	Total	Final pathologic N category for axilla	
		ypN0/ypN0i+	ypN1mi-N3
Negative Isolated tumor cells Microscopic Macroscopic	28 3 2 21	27 1 0 0	1 2 2 21

## Clipped Axillary Node as an Indicator Node (Fig. 2)

**Fig. 2:**
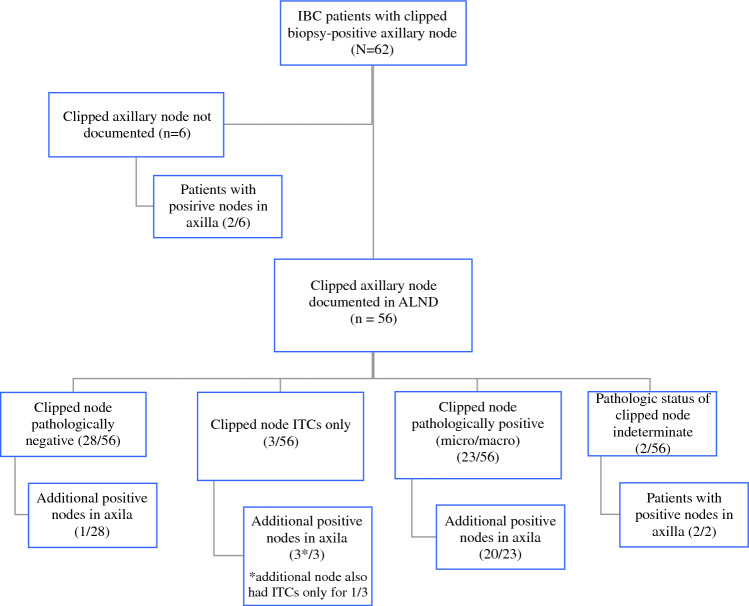
Flow chart of inflammatory breast cancer (IBC) patients with clip placement in the positive axillary node at initial diagnosis

Of 62 patients with IBC that had a clip placed in the biopsy-positive node, the clip was documented as retrieved within the axillary node dissection in 90% (56/62) patients. In 6 patients, clip removal could not be confirmed. Among the 56 with confirmed retrieval of the biopsy-positive clipped node, the clipped node was pathologically negative for any invasive tumor cells (no ITCs or greater) in 50% (28/56), which accurately predicted negative status of overall axilla in 96% (27/28), while 1/28 (4%) had one additional axillary node with micrometastasis despite the negative clipped node (Fig. 2). There were 3/56 (5%) patients with ITCs only in the clipped biopsy-positive node. On pathologic examination of the axilla, one patient had 1 additional axillary node with ITCs only (ypN0i+) and the other two patients had 9 and 1 additional positive axillary nodes with macrometastasis, respectively. Of the 41% (23/56) patients with positive clipped node containing micrometastasis or greater, 87% (20/23) had additional positive axillary node(s) with the majority being macrometastases (19/20). The pathologic status of the clipped node was unable to be determined in 2/56 (4%); both of these patients had many nodes positive with macrometastasis and micrometastasis (1 ypN2, 1 ypN3).

The pathological status of clipped axillary node accurately predicted overall axillary nodal status with excellent specificity, sensitivity, and minimal FNR, whereas all post-NAC imaging and the combined clinical assessment of post-NAC ycN status showed poor predictive ability with FNRs of 50% or greater (Table 3).

**Table 3:** Surrogates for axillary nodal response to neoadjuvant chemotherapy among patients with *cN+ IBC*

	Final axillary pathologic N category		Sensitivity		FNR (1 – Sensitivity)		Specificity	
	ypN0/ypN0i+	ypN1-3	Estimate	95% CI	Estimate	95% CI	Estimate	95% CI
US (*n* = 50)	20	30	40% (12/30)	[25–58%]	60% (18/30)	[42–75%]	65% (13/20)	[43–82%]
MRI (*n* = 50)	22	28	50% (14/28)	[33–67%]	50% (14/28)	[33–67%]	82% (18/22)	[61–93%]
PET (*n* = 37)	19	18	33% (6/18)	[16–56%]	67% (12/18)	[44–84%]	89% (17/19)	[69–97%]
Clinical (ycN) nodal status (*n* = 88)	45	43	40% (17/43)	[26–54%]	60% (26/43)	[46–74%]	76% (34/45)	[61–86%]
Clipped node micromets or greater (*n* = 54)	28	26	88% (23/26)	[71–96%]	12% (3/26)	[4–29%]	100% (28/28)	[88–100%]
Clipped node ITCs or greater (*n* = 54)	28	26	96% (25/26)	[81–99%]	4% (1/26)	[1–19%]	96% (27/28)	[82–99%]

*FNR* false negative rate, *ITCs* isolated tumor cells, *MRI* magnetic resonance imaging, *PET* positron emission tomography, *US* ultrasound

The 28 patients for whom the clipped node was negative included 9 patients with HR+/HER2- disease, 15 patients with HER2+ disease, and 4 patients with TNBC. Using the criterion of ITCs or greater in the clipped node, the one patient (1/28) falsely classified with a negative clipped node in a ypN+ axilla had HER2+ disease. There was no substantial difference in the FNR estimate by tumor subtype: 0% (95% CI 0–30%) for HR+/HER2−, 7% (95% CI 1–30%) for HER2+ disease, and 0% (95% CI 0–50%) for TNBC, with very small sample sizes and resulting wide confidence intervals.

## Discussion

To our knowledge, our study is the first to show the utility of the clipped biopsy-positive axillary node in IBC as a reliable indicator of the overall pathologic status of the axillary nodal basin post-NAC. SLNB has not been successful in IBC due to unreliable lymphatic mapping, but this obstacle can be overcome by retrieving the clipped node. These nodes were positive at diagnosis, clipped for reference and evaluated after NAC within the context of ALND. Interestingly, the index clipped node accurately predicted nodal status of the remaining axillary nodes with a FNR of 4% by considering any level of axillary nodal disease burden. The majority of patients with IBC were HR+/HER2− and HER2+ tumors, and the nodal pCR was highest with HER2+ IBC. Our study provides strong evidence to consider node clipping and targeted resection after NAC and opens the door for considering potential omission of ALND in the event of a nodal pCR. Our findings could help to reduce arm morbidity in patients with IBC, as with newer systemic and dual anti-HER2 therapy many patients with IBC achieve nodal pCR.^4^ As the evidence is accumulating regarding the non-inferior oncological outcome of de-escalating ALND in non-IBC with nodal pCR post-NAC,^14^ identifying IBC with nodal pCR by evaluating the clipped node can possibly benefit those patients by avoiding ALND.

ALND is the current standard for IBC although it may not provide any survival benefit. In a study from the National Cancer Data Base (NCDB) of about 3500 IBC cases, nodal pCR occurred in 50% of IBC cases.^3^ Dissection of > 10 nodes was taken as a proxy for ALND, and that ALND did not significantly improve survival was for cN0 and cN1 patients with IBC regardless of ypN status. Similarly, there was no survival advantage of radiotherapy in the cN0 group. A few drawbacks of the NCDB study were the lack of uniform diagnostic criteria for IBC from the heterogenous population, lack of axillary node biopsy at presentation for all patients, and lack of breast-specific survival. Nevertheless, these findings suggest that selective ALND in IBC may be reasonable to consider.

The effort to deescalate ALND in noninflammatory node-positive BCs has expanded the possibilities for SLNB post-NAC.^10^ As the FNR in ACOSOG Z1071 was 12.6% in cN1 patients with two or more SLN evaluated,^10^ cases with retrieval of clipped nodes had a lower FNR of 6.8%.^11^ This led to subsequent recommendations for intentional (“targeted”) removal of the clipped node, combined with dual tracer sentinel node technique, resulting in an even lower FNR of 4.2%.^15^ More recently, targeted removal of clipped nodes has been questioned, as in one report the clipped nodes were confirmed as sentinel nodes in up to 88% of BCs post-NAC,^16^ and there were no differences in local recurrence when three sentinel nodes were removed, regardless of retrieving the clipped node. However, the scope of SLNB and targeted SLNB in these studies was limited to patients without IBC. There is an ongoing clinical trial (NCT04636710) on dual tracer SLNB in IBC (retrieval of clipped nodes is encouraged but not required), and results will advance knowledge about the efficacy of lymphoscintigraphy and SLNB in IBC.^17^

The current study presents a straightforward approach with a technique familiar to most surgeons by focusing on retrieval of the clipped node in IBC post-NAC. Our approach of clipping a suspicious axillary node at diagnosis prior to systemic therapy is in line with the National Comprehensive Cancer Network’s guideline to promote removal of biopsy-positive lymph nodes during axillary dissection.^18^ Of note, our study on IBC also shows that the pathologic status of the clipped node is helpful in cN2 and cN3 disease as well, whereas this remains unclear in the post-NAC SLNB studies in patients without IBC that mainly focused on cN1 disease.^11,16^ At present, the American Society of Breast Surgeons recommends ALND for all cN2-3 post-NAC.^19^

The similar histology, clinical nodal status, axillary nodal involvement, and tumor biology in clipped and non-clipped patients with IBC in our study argues against substantial selection bias regarding which patients had clips placed in positive nodes. Retrieval of the clipped axillary node after NAC can be improved by localizing the clipped node preoperatively with an ^125^I seed.^20^ Only 13% of our patients had seed localization and this could be routinely implemented to increase the retrieval of clipped nodes. More than 50% of our patients with IBC with clipped nodes attained axillary pCR and they could have potentially avoided ALND. Furthermore, the FNR for a clipped axillary node was 4% in our study after considering ITCs in the clipped node as positive. The only patient with a falsely negative clipped node had a microscopic disease in a non-clipped node. Interestingly, even a clipped node with ITCs was reflective of macroscopic nodal disease in non-clipped nodes for two of three patients in our study. Excluding ITCs in the clipped node increased the FNR to 12% in our patients. Similar findings have been reported in a large prospective multicenter study of patients who did not have IBC where inclusion of ITCs in sentinel node after NAC resulted in FNR of 9% but that increased to 14% if ITCs were not considered positive.^21^ This finding is also supported by poor prognosis of BCs with ITCs post-NAC in patients who do not have IBC.^22^ In our study, the majority of the patients with IBC (either clipped/non-clipped) had level 1 and 2 axillary nodal involvement. This represents feasibility in clipping and retrieval of these nodes.

Our study paves the way for longer-term oncological and survival advantage of selectively avoiding ALND in about 50% of patients with IBC who attain nodal pCR that can be confirmed by retrieving the clipped node post-NAC. As chemotherapy regimens improve and more pCR responses are identified, there is potential for omitting ALND in the setting of IBC. The nodal pCR was higher with HR−/HER2+ IBC. Similar higher axillary nodal pCR has been reported in larger studies of IBC^4^ and has been related to survival advantage.^23,24^ Moreover, axillary nodal pCR is associated with greater survival advantage than breast pCR in non-IBC,^25,26^ thus highlighting the significance of achieving nodal pCR and accurately predicting it via less extensive axillary staging procedure, such as by evaluation of a biopsy-positive clipped axillary node after NAC as demonstrated by our study. The significance of residual axillary disease post-NAC in IBC has not been clearly defined but may be similar to finding in non-IBC studies, where residual axillary disease portends a worse prognosis and likely likely drive systemic therapy recommendations shown to improve outcomes for patients with residual disease in the setting of non-IBC.^25,26^

Preoperative imaging after NAC is often used to assess treatment response, but in this study imaging results correlated poorly with the final pathologic status of the axillary nodes. We found that the clipped node was a much stronger predictor of post-NAC nodal status in IBC. Similarly, poor yield of post-NAC imaging in predicting overall axillary nodal status has been shown in non-IBC and hence axillary staging remains a mandatory component of surgery in clinically node-positive breast cancer.^27–30^

The strength of this study lies in being one of the first studies to highlight the importance of the clipped axillary node in accurately predicting the final pathological nodal status post-NAC in patients with IBC, and furthermore the superiority of the clipped node compared with preoperative post-chemotherapy imaging in reflecting the final axillary pathologic status. Given the rarity of IBC and the single-institution experience, the sample size is substantial but validation in other larger and multi-institutional datasets will strengthen these findings. The study is limited by its retrospective nature, lack of racial diversity in our sample, varying practice patterns in the placement of clips over time, and possible selection bias regarding which patients did versus did not have axillary clips placed. However, the findings that patients with and without clips showed no significant differences on key clinical features and the trend we observed of increasing and near universal clipping of positive nodes at diagnosis demonstrates this as a routine component of our practice, reducing likelihood of substantial bias. Our study also cannot address the added advantage of targeted axillary dissection with dual tracer mapping, as axillary mapping with dual tracers was not performed in these patients.

## Conclusions

Here we provide evidence that the pathologic status of the clipped axillary node following NAC among patients with IBC is a highly accurate indicator of the status of the axillary nodal basin with a low false negative rate of 4%. These data support testing whether a pCR in the clipped axillary lymph node might permit omission of axillary lymph node dissection in selected patients with IBC. Larger-scale validation of this work is planned. Evaluation of the oncologic outcomes of a surgical de-escalation strategy for patients with IBC remains to be investigated.

## Supplementary Information

Below is the link to the electronic supplementary material.

Supplementary file1 (DOCX 19 kb)

## References

[1] Walshe JM, Swain SM. Clinical aspects of inflammatory breast cancer. *Breast Dis*. 2005-2006; 22:35-44. 10.3233/bd-2006-22105 16735785

[2] Farley CR, Irwin S, Adesoye T, et al. Lymphedema in inflammatory breast cancer patients following trimodal treatment. *Ann Surg Oncol*. 2022;29(10):6370–8. 35854031 10.1245/s10434-022-12142-7

[3] Fayanju OM, Ren Y, Greenup RA, et al. Extent of axillary surgery in inflammatory breast cancer: a survival analysis of 3500 patients. *Breast Cancer Res Treat*. 2020;180(1):207–17. 31960171 10.1007/s10549-020-05529-1 PMC7050768

[4] Kupstas AR, Hoskin TL, Day CN, Boughey JC, Habermann EB, Hieken TJ. Biological subtype, treatment response and outcomes in inflammatory breast cancer using data from the National Cancer Database. *Br J Surg*. 2020;107(8):1033–41. 32057107 10.1002/bjs.11469

[5] Stearns V, Ewing CA, Slack R, Penannen MF, Hayes DF, Tsangaris TN. Sentinel lymphadenectomy after neoadjuvant chemotherapy for breast cancer may reliably represent the axilla except for inflammatory breast cancer. *Ann Surg Oncol*. 2002;9(3):235–42. 11923129 10.1007/BF02573060

[6] DeSnyder SM, Mittendorf EA, Le-Petross C, et al. Prospective feasibility trial of sentinel lymph node biopsy in the setting of inflammatory breast cancer. *Clin Breast Cancer*. 2018;18(1):e73–7. 28755879 10.1016/j.clbc.2017.06.014

[7] Donker M, van Tienhoven G, Straver ME, et al. Radiotherapy or surgery of the axilla after a positive sentinel node in breast cancer (EORTC 10981–22023 AMAROS): a randomised, multicentre, open-label, phase 3 non-inferiority trial. *Lancet Oncol*. 2014;15(12):1303–10. 25439688 10.1016/S1470-2045(14)70460-7 PMC4291166

[8] Giuliano AE, Ballman KV, McCall L, et al. Effect of axillary dissection vs no axillary dissection on 10-year overall survival among patients with invasive breast cancer and sentinel node metastasis: the ACOSOG Z0011 (Alliance) randomized clinical trial. *JAMA*. 2017;318(10):918–26. 28898379 10.1001/jama.2017.11470 PMC5672806

[9] Bartels SAL, Donker M, Poncet C, et al. Radiotherapy or surgery of the axilla after a positive sentinel node in breast cancer: 10-year results of the randomized controlled EORTC 10981–22023 AMAROS Trial. *J Clin Oncol*. 2023;41(12):2159–65. 36383926 10.1200/JCO.22.01565

[10] Boughey JC, Suman VJ, Mittendorf EA, Ahrendt GM, Wilke LG, Taback B, et al. Sentinel lymph node surgery after neoadjuvant chemotherapy in patients with node-positive breast cancer: the ACOSOG Z1071 (Alliance) clinical trial. *JAMA*. 2013;310(14):1455–61. 24101169 10.1001/jama.2013.278932 PMC4075763

[11] Boughey JC, Ballman KV, Le-Petross HT, et al. Identification and resection of clipped node decreases the false-negative rate of sentinel lymph node surgery in patients presenting with node-positive breast cancer (T0–T4, N1–N2) who receive neoadjuvant chemotherapy: results from ACOSOG Z1071 (Alliance). *Ann Surg*. 2016;263(4):802–7. 26649589 10.1097/SLA.0000000000001375 PMC4777661

[12] Kane G, Fleming C, Heneghan H, et al. False-negative rate of ultrasound-guided fine-needle aspiration cytology for identifying axillary lymph node metastasis in breast cancer patients. *Breast J*. 2019;25(5):848–52. 31197915 10.1111/tbj.13402

[13] Rautiainen S, Masarwah A, Sudah M, et al. Axillary lymph node biopsy in newly diagnosed invasive breast cancer: comparative accuracy of fine-needle aspiration biopsy versus core-needle biopsy. *Radiology*. 2013;269(1):54–60. 23771915 10.1148/radiol.13122637

[14] Giacomo Montagna, Mary Mrdutt, Astrid Botty, et al. Oncological outcomes following omission of axillary lymph node dissection in node positive patients downstaging to node negative with neoadjuvant chemotherapy: the OPBC-04/EUBREAST-06/OMA study [abstract]. In: Proceedings of the 2022 San Antonio Breast Cancer Symposium; 2022 Dec 6-10; San Antonio, TX. Philadelphia (PA): AACR; *Cancer Res* 2023;83(5 Suppl): Abstract nr GS4-02.

[15] Caudle AS, Yang WT, Krishnamurthy S, et al. Improved axillary evaluation following neoadjuvant therapy for patients with node-positive breast cancer using selective evaluation of clipped nodes: implementation of targeted axillary dissection. *J Clin Oncol*. 2016;34(10):1072–8. 26811528 10.1200/JCO.2015.64.0094 PMC4933133

[16] Montagna G, Lee MK, Sevilimedu V, Barrio AV, Morrow M. Is nodal clipping beneficial for node-positive breast cancer patients receiving neoadjuvant chemotherapy? *Ann Surg Oncol*. 2022;29(10):6133–9. 35902495 10.1245/s10434-022-12240-6 PMC10109537

[17] Nakhlis F. Refining local-regional therapy for IBC. www.ClinicalTrials.gov. ID NCT04636710. [Accessed Feb 15, 2024].

[18] NCCN Clinical Practice Guidelines in Oncology (NCCN Guidelines®). Breast Cancer. Version 5.2023 — December 5, 2023. Available at https://www.nccn.org/professionals/physician_gls/pdf/breast.pdf

[19] The American Society of Breast Surgeons. Consensus statement on axillary management for patients with in-situ and invasive breast cancer: a concise overview (breastsurgeons.org) (March, 2022).

[20] Nguyen TT, Hieken TJ, Glazebrook KN, Boughey JC. Localizing the clipped node in patients with node-positive breast cancer treated with neoadjuvant chemotherapy: early learning experience and challenges. *Ann Surg Oncol*. 2017;24(10):3011–6. 28766234 10.1245/s10434-017-6023-z

[21] Boileau JF, Poirier B, Basik M, et al. Sentinel node biopsy after neoadjuvant chemotherapy in biopsy-proven node-positive breast cancer: the SN FNAC study. *J Clin Oncol*. 2015;33(3):258–64. 25452445 10.1200/JCO.2014.55.7827

[22] Wong SM, Almana N, Choi J, et al. Prognostic significance of residual axillary nodal micrometastases and isolated tumor cells after neoadjuvant chemotherapy for breast cancer. *Ann Surg Oncol*. 2019;26(11):3502–9. 31228134 10.1245/s10434-019-07517-2

[23] Hieken TJ, Boughey JC, Degnim AC, Glazebrook KN, Hoskin TL. Inflammatory breast cancer: durable breast cancer-specific survival for HER2-positive patients with a pathologic complete response to neoadjuvant therapy. *Ann Surg Oncol*. 2022;29(9):5383–6. 35773563 10.1245/s10434-022-12037-7

[24] Hieken TJ, Murphy BL, Boughey JC, Degnim AC, Glazebrook KN, Hoskin TL. Influence of biologic subtype of inflammatory breast cancer on response to neoadjuvant therapy and cancer outcomes. *Clin Breast Cancer*. 2018;18(4):e501–6. 29089281 10.1016/j.clbc.2017.10.003

[25] Mougalian SS, Hernandez M, Lei X, et al. Ten-year outcomes of patients with breast cancer with cytologically confirmed axillary lymph node metastases and pathologic complete response after primary systemic chemotherapy. *JAMA Oncol*. 2016;2(4):508–16. 26720612 10.1001/jamaoncol.2015.4935 PMC4845895

[26] Hennessy BT, Hortobagyi GN, Rouzier R, et al. Outcome after pathologic complete eradication of cytologically proven breast cancer axillary node metastases following primary chemotherapy. *J Clin Oncol*. 2005;23(36):9304–11. 16361629 10.1200/JCO.2005.02.5023

[27] Maeshima Y, Sakai T, Ogiya A, et al. Assessment of axillary node status by ultrasound after neoadjuvant chemotherapy in patients with clinically node-positive breast cancer according to breast cancer subtype. *Sci Rep*. 2021;11(1):10858. 34035335 10.1038/s41598-021-89738-8 PMC8149690

[28] Abel MK, Greenwood H, Kelil T, et al. Accuracy of breast MRI in evaluating nodal status after neoadjuvant therapy in invasive lobular carcinoma. *NPJ Breast Cancer*. 2021;7(1):25. 33674614 10.1038/s41523-021-00233-9 PMC7935955

[29] Hieken TJ, Boughey JC, Jones KN, Shah SS, Glazebrook KN. Imaging response and residual metastatic axillary lymph node disease after neoadjuvant chemotherapy for primary breast cancer. *Ann Surg Oncol*. 2013;20(10):3199–204. 23846781 10.1245/s10434-013-3118-z

[30] Samiei S, de Mooij CM, Lobbes MBI, Keymeulen KBMI, van Nijnatten TJA, Smidt ML. Diagnostic performance of noninvasive imaging for assessment of axillary response after neoadjuvant systemic therapy in clinically node-positive breast cancer: a systematic review and meta-analysis. *Ann Surg*. 2021;273(4):694–700. 33201095 10.1097/SLA.0000000000004356

