# Supplementary material for: Clipped Axillary Node as a Potential Surrogate for Overall Axillary Nodal Status in Inflammatory Breast Cancer Patients after Neoadjuvant Chemotherapy
Source: Ann Surg Oncol. 2024 Aug 9;31(11):7431–40. doi: 10.1245/s10434-024-15796-7 (PMC11452512; doi:10.1245/s10434-024-15796-7)
Supplement: Supplementary file 1 — Supplementary file1 (DOCX 19 kb) [file 10434_2024_15796_MOESM1_ESM.docx]

**Supplementary Table 1. Pre- and post-neoadjuvant chemotherapy imaging features of n=90 cN+ inflammatory BCs overall and comparing patients with vs without a pre-treatment biopsy-positive clipped node.**

|  |  | |  |  |  |  |
| --- | --- | --- | --- | --- | --- | --- |
|  | | **Total (N=90)** | | **Clipped Biopsy+ Node (N=62)** | **No Biopsy+ Clipped Node (N=28)** | **P-value** |
|  | |  | |  |  |  |
| **Pre-NAC Ultrasound/Mammogram performed**, n (%) | |  | |  |  | >0.99 |
| No | | 3 (3.3%) | | 2 (3.2%) | 1 (3.6%) |  |
| Yes | | 87 (96.7%) | | 60 (96.8%) | 27 (96.4%) |  |
|  | |  | |  |  |  |
| **Pre-NAC MRI performed**, n (%) | |  | |  |  | 0.06 |
| No | | 13 (14.4%) | | 12 (19.4%) | 1 (3.6%) |  |
| Yes | | 77 (85.6%) | | 50 (80.6%) | 27 (96.4%) |  |
|  | |  | |  |  |  |
| **Pre-NAC PET performed**, n (%) | |  | |  |  | 0.73 |
| No | | 11 (12.2%) | | 7 (11.3%) | 4 (14.3%) |  |
| Yes | | 79 (87.8%) | | 55 (88.7%) | 24 (85.7%) |  |
|  | |  | |  |  |  |
| **Internal Mammary Node Suspicious by Imaging (Pre-NAC)**, n (%) | |  | |  |  | 0.60 |
| No | | 68 (75.6%) | | 48 (77.4%) | 20 (71.4%) |  |
| Yes | | 22 (24.4%) | | 14 (22.6%) | 8 (28.6%) |  |
|  | |  | |  |  |  |
| **Supraclavicular Node Suspicious by Imaging (Pre-NAC)**, n (%) | |  | |  |  | 0.25 |
| No | | 74 (82.2%) | | 53 (85.5%) | 21 (75.0%) |  |
| Yes | | 16 (17.8%) | | 9 (14.5%) | 7 (25.0%) |  |
|  | |  | |  |  |  |
| **Infraclavicluar Node Suspicious by Imaging (Pre-NAC)**, n (%) | |  | |  |  | 0.18 |
| No | | 78 (86.7%) | | 56 (90.3%) | 22 (78.6%) |  |
| Yes | | 12 (13.3%) | | 6 (9.7%) | 6 (21.4%) |  |
|  | |  | |  |  |  |
| **Post-NAC Imaging Nodal Response (Ultrasound)**, n (%) | |  | |  |  | 0.07 |
| Complete Response | | 30 (60.0%) | | 23 (69.7%) | 7 (41.2%) |  |
| Partial or No Response | | 20 (40.0%) | | 10 (30.3%) | 10 (58.8%) |  |
| Missing | | 40 | | 29 | 11 |  |
|  | |  | |  |  |  |
| **Post-NAC Imaging Nodal Response (MRI)**, n (%) | |  | |  |  | 0.14 |
| Complete Response | | 31 (62.0%) | | 23 (69.7%) | 8 (47.1%) |  |
| Partial or No Response | | 19 (38.0%) | | 10 (30.3%) | 9 (52.9%) |  |
| Missing | | 40 | | 29 | 11 |  |
|  | |  | |  |  |  |
| **Post-NAC Imaging Nodal Response (PET)**, n (%) | |  | |  |  | >0.99 |
| Complete Response | | 30 (78.9%) | | 21 (77.8%) | 9 (81.8%) |  |
| Partial or No Response | | 8 (21.1%) | | 6 (22.2%) | 2 (18.2.0%) |  |
| Missing | | 52 | | 35 | 17 |  |
